# Malignant Pleural Mesothelioma: Current Understanding of the Immune Microenvironment and Treatments of a Rare Disease

**DOI:** 10.3390/cancers14184415

**Published:** 2022-09-11

**Authors:** John Tedesco, Mark Jaradeh, Wickii T. Vigneswaran

**Affiliations:** Department of Thoracic and Cardiovascular Surgery, Loyola University Medical Center, Maywood, IL 60153, USA

**Keywords:** mesothelioma, pleura, immune microenvironment, outcomes

## Abstract

**Simple Summary:**

Malignant pleural mesothelioma is a rare disease with an annual incidence of around 3000 cases a year in the United States. Most cases are caused by asbestos exposure, with a latency period of up to 40 years but overall survival of approximately only 6–12 months after the time of diagnosis. Often, the treatment is multimodal and consists of surgery, chemotherapy, and radiation. While the survival benefit of treatment is impactful, overall prolongation remains marginal. Nevertheless, the advent of new treatment approaches involving the interactions of targeted immune therapies and the tumor microenvironment appear to offer some promise. Furthering our understanding of these complex interactions in conjunction with the host immune system will likely prove to be pivotal in advancing current treatment options for malignant pleural mesothelioma.

**Abstract:**

Malignant pleural mesothelioma is a rare disease with an annual incidence of around 3000 cases a year in the United States. Most cases are caused by asbestos exposure, with a latency period of up to 40 years. Pleural mesothelioma is an aggressive disease process with overall survival of roughly 6–12 months after the time of diagnosis. It is divided into three subtypes: epithelioid, mixed type, and sarcomatoid type, with the epithelioid subtype having the best overall survival. Often, the treatment is multimodality with surgery, chemotherapy, and radiation. The survival benefit is improved but remains marginal. New treatment options involving targeted immune therapies appear to offer some promise. The tumor microenvironment is the ecosystem within the tumor that interacts and influences the host immune system. Understanding this complex interaction and how the host immune system is involved in the progression of the disease process is important to define and guide potential treatment options for this devastating and rare disease.

## 1. Introduction

Malignant pleural mesothelioma (MPM) is a rare disease process that arises from mesothelial membranes and often affects the pleura and peritoneum. The pathophysiology of the disease process is intimately linked to asbestos exposure, with a latent period of about 40 years prior to presentation. When it involves the pleura, which is the commonest type, patients present with dyspnea, chest pain, fatigue, or weight loss. On imaging, there is often a pleural effusion with associated pleural thickening or a pleural-based mass. It is a highly aggressive tumor, and even with current treatment regimens, the overall median survival is around 17 months, and the 5-year overall survival is about 10%. Due to the regulated use of asbestos in the United States, the incidence of MPM is decreasing; however, in other countries such as China, Russia, and Western Europe, the incidence of MPM is on the rise [1].

Patients who are deemed resectable are typically treated with trimodality therapy with chemotherapy, surgery, and radiation therapy. Unfortunately, most patients present with advanced-stage disease and are unable to be offered surgery and are treated with first-line therapy with cisplatin/pemetrexed chemotherapy. Despite advances in surgical technique, chemotherapy, and delivery of radiation, the mean survival benefit for resectable disease remains marginal [2,3].

One area of interest that has revolutionized cancer therapy has been the discovery of immune checkpoint inhibitors. For example, the use of nivolumab and ipilimumab in other types of cancers such as melanoma, non-small-cell lung cancer, and renal cell carcinoma have shown promise in enhancing the antitumor function of T-cell responses and, when used in combination with traditional chemotherapy regimens, have shown a survival benefit [4,5].

This paper is a comprehensive review of the literature on our current understanding of the pathophysiology, complex interaction of the tumor microenvironment with the host immune response, and how this may translate to potential therapeutic targets in the treatment of MPM

## 2. Epidemiology

MPM is a rare tumor with an annual incidence of 3000 cases per year in the United States. More than 80% of mesothelioma cases are due to asbestos exposure, with a latency period of up to 40 years after exposure. Other risk factors include Simian Virus 40 (SV40) infection, prior chest wall radiation, and genetic factors such as a mutated BRCA1 associated protein 1 (BAP1) gene. MPM is more common in males, and despite increased regulation on asbestos, rates have not decreased over the past 30 years. This is likely due to prior exposure in the 1970s and the latency period of the disease process that manifests in the decades after exposure. Currently, due to government regulatory efforts asbestos exposure and industrial use have substantially declined in the US. In contrast, worldwide, due to the lack of regulations, industrial mining of asbestos, and continued exposure, epidemiologic studies suggest that the incidence of MPM is continuing to increase [1,6,7,8].

MPM is an extremely aggressive tumor that originates in the serosal surfaces of the pleura, peritoneum, pericardium, and tunica vaginalis where mesothelial membranes are. It is classified histologically into three subtypes: epithelioid, biphasic/mixed type, and sarcomatoid type. The epithelial subtype is the most common subtype, representing approximately 50% of cases, and is associated with the best overall prognosis. The sarcomatoid subtype makes up about a quarter of cases and is associated with poor prognosis. A recent population study from the national cancer database of over 19,000 patients with MPM demonstrated that patients with sarcomatoid histology have locally advanced disease at the time of presentation. On multivariable analysis, sarcomatoid and its desmoplastic subvariant and biphasic/mixed subtype histology were independent predictors of worse survival. Notably, desmoplastic malignant mesothelioma, a subvariant of sarcomatoid type, is characterized histologically by dense stromal fibrosis and has the worst prognosis overall. Other clinical variables that are associated with poor prognosis include poor performance status (inability to perform 6 min walk test, ECOG score 3 or more), age 75 or more, government insurance, median income less than USD 63,000, tumor stage, tumor volume, and elevated LDH [6,7].

## 3. Pathophysiology

The pathophysiology of MPM is extremely complex with multiple cellular and environmental interactions, all of which appear to be linked to a chronic inflammatory state, ultimately leading to malignant mesothelial cell transformation, proliferation, and a unique tumor microenvironment. Most cases of MPM are due to occupational exposure to asbestos, followed by an intense immune response leading to malignant proliferation. Typically, it is seen in workers who have had many years of high-level occupational exposure. Studies have shown an exposure dose threshold of 25 to 100 fibers/mL/yr significantly increases the risk of developing MPM, and the latency period is inversely proportional to exposure level. Asbestos is a unique crystalline molecule that lends itself to inducing a robust and protracted immune response with excessive cellular proliferation and collagen deposition. The geometry and dimensions of each subtype may govern their deposition and clearance kinetics, biological reactivity, and dissolution in the lung. Furthermore, the chemical composition and surface properties, including absorption, oxidation/reduction reactions, and charge, also play a role in biopersistence, cellular responses, and pathogenicity. Importantly, smoking seems to have a synergistic effect on the pathogenesis of MPM. Some evidence suggests that smoke exposure increases the rate of asbestos fiber retention, thus promoting and exacerbating the effects of asbestos. Typically, asbestos fibers will cause a diffuse interstitial fibrosis in the lower lung zones, with worse disease closest to the pleura and honeycombing of the lung in advanced cases. Microscopically, the disease process is defined by diffuse interstitial fibrosis and the presence of asbestos bodies [6,7].

The initial insult begins when mesothelial cells encounter asbestos fibers and generate multiple macrophage attractants (CCL2, IL-6, IL-8, macrophage inflammatory protein-1, granulocyte colony-stimulating factor, and granulocyte/macrophage colony-stimulating factor), which begins the inflammatory cascade. In vitro and in vivo studies have demonstrated that upon exposure to asbestos, macrophages attempt to phagocytose asbestos fibers but, due to their size, are unable to do so. This phenomenon of the “frustrated macrophage” then triggers a cytokine cascade and formation of reactive oxygen species that promote a persistent proinflammatory state, which eventually leads to DNA damage, gene deletions, and tissue hypoxia. In addition, the alveolar macrophage (AM) response promotes a chronic inflammatory state, induces fibrosis, and upregulates the expression of genes linked to cellular proliferation and collagen deposition [6].

Macrophages are also responsible for allowing damaged mesothelial cells that should normally be targeted for apoptosis to evade the immune system. Cells that have sustained genomic insult are ordinarily marked for poly(ADP)ribose polymerase-induced programmed cell death but, under certain signaling influences, are rescued from being terminated by aspects of the inflammatory response. In vitro experiments have shown that increased levels of TNF-α from persistently activated macrophages upregulate the NF-κβ pathway and subvert mesothelioma cells from programmed cell death [6].

Finally, a growing body of research into cytogenetics and molecular genetics has explored new insights into the pathogenesis of this malignancy. Recent evidence suggests that the extracellular matrix (ECM) may have implications in the pathogenesis of MPM, in which the surrounding stroma promotes tumor growth, invasion, and protection from the antitumor response. Many genes related to the synthesis of and interaction with the extracellular matrix (ECM) are upregulated in patients with MPM, which help promote a protumor environment. The more aggressive forms of MPM (biphasic/mixed subtype, desmoplastic and sarcomatoid) are associated with upregulation of matrix metalloproteases (MPPs), which promote cellular invasion [2,5].

## 4. Tumor Microenvironment: Cellular Makeup and Molecular Signaling

Our understanding of the immune system and its interaction with cancer cells has unfolded over the last 20 years, leading to a whole field study and investigation into the intricate relationship between tumors and immune cells. The tumor microenvironment is the complex and fluid interaction of proliferating tumor cells, extracellular matrix, nutrients, cytokines, and immune cells, specifically, tumor-associated macrophages (TAMs) and tumor-infiltrating lymphocytes (TILs), that help promote tumor growth and metastases. Understanding this complex relationship has led to the development of new treatments in multiple solid-organ tumors and may offer potential drug targets in the treatment of MPM. The immune system is instrumental in assessing the host environment for potential threats. Tumor surveillance begins with “immune editing”, which describes the phenomena of an immune competent host developing cancer in the setting of active immunosurveillance. It is divided into three phases: elimination, equilibrium, and escape. During the elimination phase, the host immune system is upregulated, and host mechanisms can induce apoptosis of tumor cells. If this process is unsuccessful and tumor cells are not fully eradicated, then the tumor will enter the equilibrium phase. This phase is defined by tumor growth and maintenance, which eventually will lead to disease progression. In the escape phase, malignant cells will adapt to the host immune environment and go on to further develop tumor variants that can circumvent the cytotoxic capabilities of the host immune system and eventually lead to tumor metastasis [7,9].

Macrophages are typically one of the first immune cells involved in the initial response to antigens and have a myriad of functions. They are derived from monocytes and after several stages of development within the bone marrow are released into peripheral circulation and migrate into resident tissues and differentiate into macrophages. They have tremendous plasticity and, depending on the tumor microenvironment, can support or combat tumor cells. Local cytokine production and ligands will stimulate macrophages to either M1 or M2 tissue-associated macrophages (TAMs). The Ujiie study found that TAMs (CD163+ macrophages) and their ratio with biologically relevant TILs (CD8 and CD20 lymphocytes) were independent predictors of survival in epithelioid MPM. In patients that had not undergone neoadjuvant chemotherapy, high stromal CD163+ TAMs/M2 tumor-associated macrophages were associated with poor survival. Interestingly, it appears that CD163+ TAMs secrete immunosuppressive cytokines and support tumor progression, invasion, angiogenesis, and metastases. The interaction with MPM cells appeared to shift mature macrophages toward the M2 phenotype, which is characterized by poor antigen presentation and increased immunosuppressive activity. Studies have shown that when macrophages are exposed to MPM cells, they produce higher amounts of prostaglandin E2, an arachidonic acid metabolite, which has been shown to stimulate the development of regulatory T cells (Tregs), which in turn will downregulate the host T-cell response. Furthermore, some evidence suggests that TAMs can upregulate IL-10 and B7-H3 on tumor cells, both of which are known to downregulate the immune response and inhibit antitumor T-cell responses [10,11].

B lymphocytes (CD20+), a component of the adaptive immune response, are also fundamental in mounting an effective host immune response and have multiple roles in the immune system. TAMs produce stimulatory signals to B lymphocytes, leading to migration into the tumor microenvironment. Once in tissues, they can function as antigen-presenting cells and provide costimulatory signals to T cells, or they can differentiate into antibody-secreting plasma cells. Previous studies have established that higher expression of tumor-infiltrating CD20 B lymphocytes in the tumor microenvironment has been associated with improved patient survival in primary breast cancer, non-small-cell lung cancer, epithelioid ovarian cancer, and pancreatic cancer. The Ujiie study demonstrated some evidence that B cells may have a role in constraining epithelioid MPM. However, this finding remains controversial and inconsistent in other in vivo studies. Additionally, murine models have demonstrated that B cell infiltration may potentiate the chronic inflammatory state and could possibly enhance tumor development and progression [10,11,12].

T cells are part of the adaptive immune response. They originate in the bone marrow and then migrate to the thymus gland and mature into distinct cell lines (CD4+, CD8+, regulatory T cells) with various functions. CD8+ “killer T cells” use T-cell receptors (TCRs) to recognize antigenic peptides bound to MHCI molecules on the surface of cells infected with viruses or mutated cancer cells and induce apoptosis. They also produce tumor necrosis alpha (TNFα) and interferon-gamma (IFNγ). The Ujiie study also showed that although not statistically significant, a high density of CD8 TILs in tumors tended to exhibit an improved overall survival in patients with MPM. Anraku et al. performed an immunohistochemical analysis of 32 patients who had undergone extrapleural pneumonectomy to assess the distribution of helper, cytotoxic killer, and regulatory T cells. Based on their multivariate analysis, they demonstrated that patients who expressed high levels of CD8+ TILs conferred a more favorable overall survival, disease-free progression, and reduced frequency of lymph node metastases than in patients with higher expression of regulatory and CD4+ T cells. [11,12].

Cancer tissues are diverse and composed of various types of cells with distinct molecular and phenotypic features. Malignant cells are adaptable and due to the changing environment and immune response, they can differentiate into subclones and evolve. Understanding intratumoral heterogeneity and the relationship to the tumor microenvironment is clinically important because it could potentially impact therapies. Kiytoani and colleagues completed multiregional DNA sequencing on six patients from geographically different regions of MPM tumors (anterior, posterior, and diaphragm) and characterized somatic mutations within each region, mutation/neoantigen load, spatial heterogeneity of somatic mutations of cancer cells, and tumor-infiltrating lymphocytes. Their analysis identified distinct patterns of somatic mutations and immune microenvironment signatures (TCRβ repertoires) and immune microenvironment both intratumorally and between each patient. In their study, they identified the active and suppressive sides of the tumor immune microenvironment that can coexist at the same time. They demonstrated that higher cytolytic activity, represented by the PRF1/TRB ratio in tumor sites, correlated with higher numbers of somatic mutation/neoantigen load, and more robust expansion of TILs. However, they also found that the FOXP3/TRB ratio, which represents Treg activity, was also higher within tumor positions with higher mutation/neoantigen load, and that these areas also expressed lower diversity of TILs. This indicates a balance between immune cell activation and inhibition within the tumor microenvironment, and that once CD8+ TILs are activated and try to eradicate tumor cells, immune suppressive molecules and Tregs may respond and inadvertently assist cancer cells in escaping the host immune system [13].

Investigations into immune system cytokine pathways have also expanded our knowledge of the communication and signaling pathways. Another component of the tumor immune microenvironment is the mesothelioma secretome and metabolome, both of which promote chemotaxis and cellular differentiation through chemokines, growth factors, and metabolites. The mesothelioma secretome includes the chemokines CCL2, CCL4, CXCL10, CXCL5, CXCL1, and CXCL12, the cytokines IL-10, IL-6, and growth factors TGFB, VEGF, MCSF, GM-CSF, G-CSF, FGF, and PDGF. Hypoxia is one of the cardinal features of the metabolome and can promote tumor evasion from the host immune system and enhance the growth of mesothelioma cell lines. Specifically, hypoxia induces upregulation of PD-L1 expression in tumor cell lines, which in turn downregulates the host immune system. Other examples of upregulation of gene expression in mesothelioma cells include glucose transporter 1 (Glut1) receptors and L-type amino acid transporter 1 (LAT1), both of which provide a competitive advantage for nutrients to tumor cells over the host immune system [9].

One cytokine that has been identified as an important immune regulatory agent is interlukin-7 (IL-7). IL-7 is essential for the development and homeostatic maintenance of T and B lymphocytes. Binding to the IL-7 receptor activates multiple pathways that regulate lymphocyte survival, proliferation, and differentiation. Studies have shown that patients with high tumor expression levels of interleukin-7 receptor (IL-7R) are associated with poor prognosis and upregulation of regulatory T cells (T regs). Studies in other cancers have demonstrated that elevated levels of Tregs in tumor beds and peripheral blood samples predict poor survival, and this has also been shown to correlate in patients with MPM. In breast and lung cancer, IL-7 has also been shown to upregulate vascular endothelial growth factor, promoting angiogenesis and tumor growth. It has been postulated that IL-7/IL-7R signaling may promote tumor growth by two separate signaling pathways, angiogenesis and the upregulation of Tregs, thereby decreasing cytotoxic T-cell activity. Therefore, the development of therapeutic targets on the IL-/IL-7R signaling axis may provide an attractive option for drug development [14].

## 5. Current and Future Treatments

MPM is a highly aggressive pleural malignancy with a 5-year mortality of more than 90%. Median survival with no treatment is approximately 7 months. Patients with stage I–IIIA disease are treated with combination therapy of surgery, chemotherapy, and radiation. The timing of each treatment modality is still debated, but typically, patients who are deemed a fit surgical candidate are offered surgery first, followed by adjuvant chemotherapy and radiation. Surgical resections for patients with MPM can either be pleurectomy with decortication (PD) or extrapleural pneumonectomy (EPP). However, due to the lack of randomized clinical trials, the choice of surgery for patients with MPM is controversial. For instance, the Mesothelioma and Radical Surgery (MARS) trial assessed whether patients treated with induction chemotherapy and then randomized to an EPP surgical arm vs. observation alone showed a high rate of surgical mortality and that EPP showed no benefit for overall survival. Notably, this study has been highly criticized due to the small sample size of only 50 patients. In contrast, the retrospective study by Yan and colleagues reported an increase in survival for select patients who underwent EPP. On their univariate analysis, surgeon experience and treatment with pemetrexed were statistically significant [1,15,16,17].

**Figure 1:**
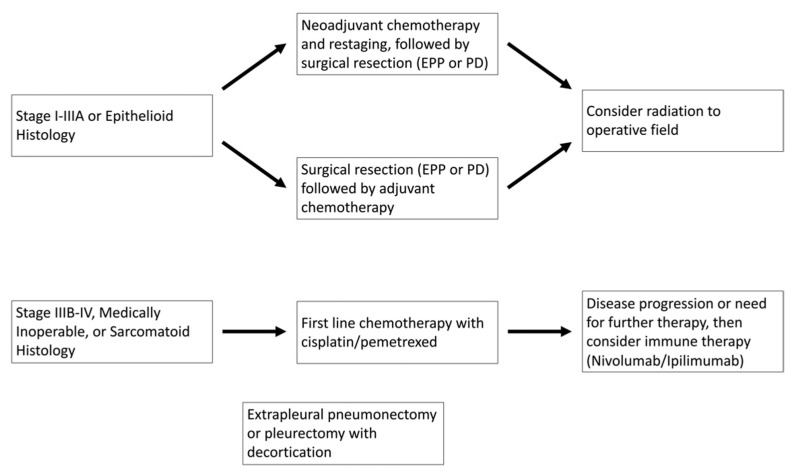
Current treatment algorithm for malignant mesothelioma. EPP, extrapleural pneumonectomy; PD, pleurectomy with decortication.

Currently, based on the results of the EMPHACIS trial, the standard of care for patients with advanced disease or who are not surgical candidates is a combination of cisplatin and pemetrexed. This randomized clinical trial included 456 patients with previously untreated mesothelioma who were not eligible for curative surgery with either combination pemetrexed and cisplatin or cisplatin alone. Their results demonstrated that the combination pemetrexed/cisplatin arm had improvements in response rate (41.3% vs. 16.7%; *p* < 0.001), median time to disease progression (5.7 vs. 3.9 months), and overall survival (12.1 vs. 9.3 months; *p* = 0.02). More recently, based on the data from the multicenter randomized clinical trial, the addition of bevacizumab to cisplatin/pemetrexed for patients with unresectable disease showed an overall increase in survival by 2.7 months when compared to chemotherapy alone (18.8 vs. 16.1 months, *p* = 0.0167), with similar adverse effect profiles [2,3]. Current treatment algorithms are summarized in Figure 1.

Due to the recalcitrant nature of the disease process, investigations into potential immunotherapies have become an attractive option. Cancer immunotherapy attempts to harness the power and specificity of the immune system to recognize and destroy tumor cells and to prevent tumor recurrence [10,18,19].

Receptor tyrosine kinases (RTKs) play a pivotal role in tumor growth and metastasis. They provide a key signal that leads to the transformation, proliferation, and invasion of tumor cells. Previous studies have shown that RTKs such as epidermal growth factor receptor (EGFR), mesenchymal/epithelial transition factor receptor (MET), insulin growth factor receptor (IGFR), and vascular endothelial growth factor receptor (VEGFR) are expressed in MPM. Notably, in mutated EGFR non-small-cell lung cancers, the hepatocyte growth factor/MET signaling pathway is associated with acquired resistance to EGFR inhibitors. Therefore, it is crucial to target MET along with complementary signaling to curtail drug resistance. Work by Kanteti and colleagues previously reported that MPM cell lines overexpress mutated MET that is constituently active, which promotes cellular proliferation. They were also able to demonstrate that the small-molecule MET inhibitor, SU11274, suppresses cell proliferation, which is now believed to potentially be a target for immune therapy. Additionally, preclinical work with tivantinib (ARQ197), a non-ATP competitive inhibitor of MET, showed it can inhibit MET in multiple cell lines, providing further evidence for the potential clinical drug application [4]

Another important downstream signaling molecule for RTKs is phosphatidylinositol 3′ kinase (PI3K). Normally, PI3K is regulated by both p110α, a catalytic subunit, and p85, a constitutively bound regulatory subunit. MPM PI3K mutations are typically overexpressed and acquire gain of function mutations, which lead to a perpetually active form of the growth signal and promote activation of downstream signaling molecules. The phosphatidyl-inositiol-3,4,5-triphosphate (PIP3) generated by the PI3K at the cell membrane recruits PDK1 and AKT, resulting in activation of mTOR complexes. The AKT and mTOR signaling cascade promotes cell proliferation and tumorigenesis. Therefore, it is more effective to simultaneously target both mTOR and PI3K. GDC-0980 and NVP-BEZ235 are new-generation small-molecule dual inhibitors of class I isoforms of PI3K and mTOR that have been shown to inhibit both molecules. Preclinical cancer models have shown that both drug compounds significantly reduce tumor growth and are currently being investigated in phase I clinical trials. [4]

Immune checkpoint inhibitors, such as anticytotoxic T-lymphocyte antigen-4 (CTLA-4) and antiprogrammed death-1 (PD-1) antibodies, have shown durable antitumor immune effects for several types of cancers. Cytotoxic T-lymphocyte-associated protein (CTLA-4) is a glycoprotein expressed by activated T cells and Tregs. CTLA-4 binds to the B7 (CD-80 and CD86) ligand that is normally expressed on antigen-presenting cells, resulting in a signaling pathway that directly inhibits T-cell effector function. Melanoma clinical trials assessing the use of CTLA-4 inhibitors have shown promise and increased overall survival. Initial studies in MPM have shown an immune-related progression-free survival was 6.2 months, and disease control was achieved in 52% of patients with a median duration of 10.9 months. The recent double-blinded, placebo-controlled, Phase 2b DETERMINE trial analyzed 569 patients with advanced-stage unresectable pleural or peritoneal malignant mesothelioma and compared tremelimumab, a CTLA-4 inhibitor, to the placebo group to the endpoint of overall survival. In their study, the 382 patients in the treatment group showed no improved OS in patients with relapsed MPM. Given these findings, more work must be completed to fully elucidate the role of CTLA-4 inhibitors in patients with MPM [7,20]. Nivolumab, a PD-1 inhibitor, and ipilimumab, a CTLA-4 inhibitor, have also shown promise in improving overall survival in patients with non-small-lung cancer, and its efficacy in MPM was recently investigated in the CheckMate 743 phase III randomized clinical trial. In this study, 605 patients with previously untreated MPM were randomized to either standard chemotherapy treatment or to nivolumab plus ipilimumab immune checkpoint inhibitors. At the prespecified interim analysis, the study demonstrated an overall survival benefit, 18.1 months versus 22.1 months (*p* = 0.002 hazard ratio of 0.74), in patients treated with immune therapy versus chemotherapy as a first-line treatment for patients with unresectable MPM. Moreover, at the 2-year follow-up, the overall survival in the immune therapy arm was greatly improved, 41% versus 27%. Based on these promising results, the National Comprehensive Cancer Network Clinical Practice Guidelines in Oncology (NCCN) now considers combination nivolumab/ipilimumab after first-line chemotherapy in patients with unresectable MPM [1,3].

Another approach in the use of immunotherapy is to use dendritic cells (DCs) to present tumor-associated antigens (TAAs), and therapy generates an immune response that specifically targets cancer cells. Dendritic-cell-based immune therapy is an attractive option because DCs are potent antigen-presenting cells (APCs) specialized for inducing activation and proliferation of CD8+ cytotoxic T lymphocytes (CTLs) and CD4 lymphocytes. A recent study by Hegman and colleagues treated 10 patients with newly diagnosed epithelial-type MPM with autologous tumor-lysate-pulsed DCs injected intradermally and documented the immunological effectiveness of the treatment. Patients first received chemotherapy and, if they showed no disease progression, went on to receive the pulsed DC tumor lysate. Recent reports have suggested that lymphocyte numbers decline after cisplatin/pemetrexed treatment, so initiating immune therapy after chemotherapy treatment may reinvigorate the immune system. In their study, nine patients did receive pemetrexed and cisplatin. One patient, due to hearing impairment, received pemetrexed and carboplatin. Serum samples of all patients were analyzed for IgG and IgM antibodies to the immunogenic protein keyhole limpet hemocyanin (KLH) were analyzed to assess the immune response. Blood samples revealed an increase in antitumor activity in four of the patients treated with DC tumor lysate. In addition, 50% of patients had induration at the skin injection site, suggesting an induced immune response. This was confirmed by increased levels of T lymphocytes and macrophages on skin biopsy samples. Finally, blood samples in six patients showed increased antitumor T-cell activity. Although this study did not show a survival benefit, it did demonstrate a host immune response to DC pulsed tumor lysate and may offer a potential therapy [19].

**Figure 2:**
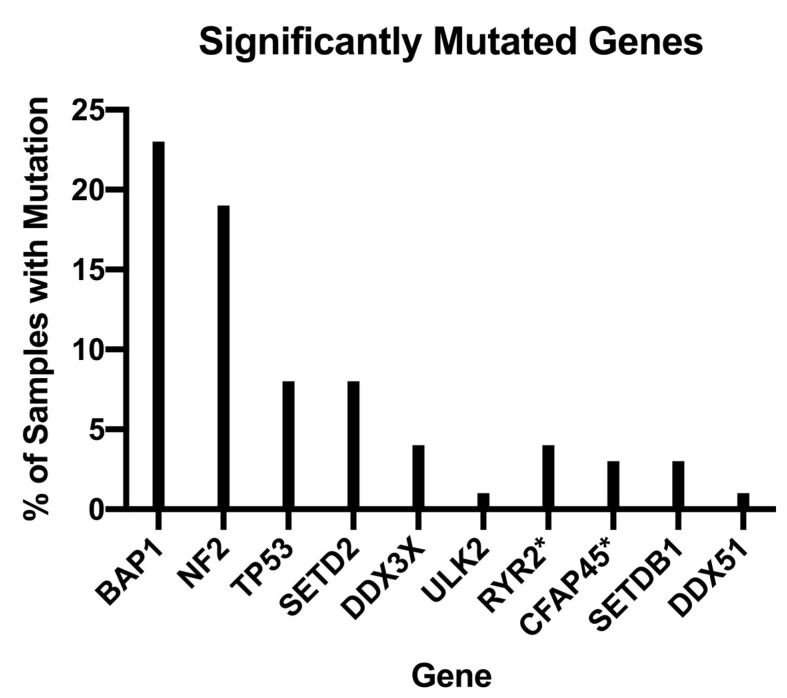
Bar graph depicting the proportion of samples studied by Bueno et al. found to have significant mutations in the corresponding gene (*n* = 202, * N = 99).

Further still, the trajectory of future treatment modalities aims to continue to personalize and tailor diagnostic and therapeutic regimens through the implementation of genetic studies and analyses. For example, in their pivotal genomic analysis of MPMs, Bueno et al. analyzed 99 whole exomes of their 216 total study population and found MPMs exhibited a myriad of genetic aberrations, notably, BAP1, NF2, TP53, SETD2, DDX3X, ULK2, RYR2, CFAP45, SETDB1, and DDX51, that demonstrated q-score ≥0.8 with a proportion of mutated samples ranging from 1 to 23%, as shown in Figure 2 [21]. In addition, Bueno et al. found recurrent mutations in SF3B1 and TRAF7, with the former showing a distinct splicing profile when compared to wild type and the latter demonstrating alterations that occurred primarily in the WD40 domain and exclusive of any observed NF2 alterations [14]. As a result of their genomic analyses, Bueno et al. found 28 total gene fusions and splice alterations that are involved in, and contribute to, the recurrent inactivation of tumor suppressors NF2, BAP1, and SETD2 [21]. The BRCA-1-associated protein (BAP1) is a tumor suppressor gene that has been implicated in the pathogenesis of uveal and cutaneous melanoma, mesothelioma, renal cell carcinoma, and breast cancer. Erber et al. further studied BAP1 by assessing its utility as an adjunct for distinguishing MM from benign tumors in their retrospective study of 306 mesothelial tumors, of which 211 were assessable for BAP1 staining. Through their studies, Erber et al. found loss of BAP1 expression demonstrated a sensitivity of 0.56, specificity of 1.00, positive predictive value of 1.00, and negative predictive value of 0.31, i.e., the loss of BAP1 expression in their cohort excluded the possibility of a benign process [22,23,24,25,26].

More recently, Girolami et al. assessed the overall diagnostic performance of a variety of biomarkers such as MTAP, p16, CDKN2A, GLUT1, IMP3, and mesothelin through a meta-analysis of 65 studies composed of 5354 patients [22,23,24]. Girolami et al. found that BAP1 alone demonstrated a specificity and sensitivity of 0.99 and 0.65, respectively, while combined BAP1 loss and homozygous deletions of p16, according to a univariate model, exhibited a specificity of 1.00 and sensitivity of 0.83, as shown in Table 1 [23]. Similarly, individual MTAP loss and p16 homozygous deletions, with the latter according to a univariate model; each demonstrated specificities of 0.99 and 1.00, respectively, and sensitivities of 0.47 and 0.62, respectively [16]. GLUT1 and IMP3 each demonstrated specificities of 0.88 and 0.90, respectively, and sensitivities of 0.82 and 0.65, respectively. Lastly, mesothelin was found to exhibit a specificity of 0.90 and sensitivity of 0.73 [23]. Thus, while recent advances in our understanding of MPM genomics potentially allow for the reactive establishment of a definitive diagnosis, further studies are needed in order to proactively identify at-risk patients through the incorporation of additional combinatorial biomarker analyses.

## 6. Conclusions

Pleural mesothelioma is an aggressive disease with dismal overall survival. Recent advances have shed insight into the pathogenesis and tumor microenvironment. This has led to new promising treatments based on the immune microenvironment. Immunotherapy is now becoming part of the treatment algorithm; however, specific targets are yet to be reliably identified. There are several treatment options on the horizon. Increasing understanding of the complex interaction of the host immune system that is involved in the progression of the mesothelioma disease process is important for future development. This knowledge will define and guide treatment options for this devastating, rare, and unique disease that is on the rise worldwide. Ongoing research in mesothelioma is important, and additional knowledge from other malignancies will also help to consider all the treatment options.

## Figures and Tables

**Table 1:** Pooled specificity and sensitivity values of biomarkers.

Marker	Specificity	Sensitivity
BAP1	0.99	0.65
BAP1 loss + p16 HD	1.00	0.83
MTAP	0.99	0.47
p16 HD	1.00	0.62
GLUT1	0.88	0.82
IMP3	0.90	0.65
Mesothelin	0.90	0.73
